# Death Associated Protein Kinases: Molecular Structure and Brain Injury

**DOI:** 10.3390/ijms140713858

**Published:** 2013-07-04

**Authors:** Syam Nair, Henrik Hagberg, Rajanikant Krishnamurthy, Claire Thornton, Carina Mallard

**Affiliations:** 1 Institute of Neuroscience and Physiology, Sahlgrenska Academy, Gothenburg University, Gothenburg 40530, Sweden; E-Mail: syam.nair@neuro.gu.se; 2 School of Biotechnology, National Institute of Technology Calicut, Calicut 673601, India; E-Mail: rajanikant@nitc.ac.in; 3 Institute of Clinical Sciences, Sahlgrenska Academy, Gothenburg University, Gothenburg 40530, Sweden; E-Mail: henrik.hagberg@obgyn.gu.se; 4 Centre for the Developing Brain, Division of Imaging Sciences & Biomedical Engineering, The Rayne Institute, King’s College London St Thomas’ Hospital, London SE1 7EH, UK; E-Mail: claire.thornton@kcl.ac.uk

**Keywords:** DAPk1, neuroprotection, neonatal hypoxia-ischemia, NMDA receptor

## Abstract

Perinatal brain damage underlies an important share of motor and neurodevelopmental disabilities, such as cerebral palsy, cognitive impairment, visual dysfunction and epilepsy. Clinical, epidemiological, and experimental studies have revealed that factors such as inflammation, excitotoxicity and oxidative stress contribute considerably to both white and grey matter injury in the immature brain. A member of the death associated protein kinase (DAPk) family, DAPk1, has been implicated in cerebral ischemic damage, whereby DAPk1 potentiates NMDA receptor-mediated excitotoxicity through interaction with the NR2BR subunit. DAPk1 also mediate a range of activities from autophagy, membrane blebbing and DNA fragmentation ultimately leading to cell death. DAPk mRNA levels are particularly highly expressed in the developing brain and thus, we hypothesize that DAPk1 may play a role in perinatal brain injury. In addition to reviewing current knowledge, we present new aspects of the molecular structure of DAPk domains, and relate these findings to interacting partners of DAPk1, DAPk-regulation in NMDA-induced cerebral injury and novel approaches to blocking the injurious effects of DAPk1.

## 1. Introduction

Perinatal brain injury is a major cause of neurological deficits in newborns leading to lifelong consequences such as cerebral palsy and delayed cognitive and behavioral deficits [1]. Perinatal asphyxia and acute encephalopathy occurs in 2–10 of 1000 live births, with a different incidence in different parts of the world [2]. The condition causes more than 800,000 deaths in the neonatal period per year worldwide and a substantial fraction of the infants that survive suffer neurological sequelae [3]. Currently, hypothermia is the only neuroprotective treatment available in term infants with acute encephalopathy that demonstrate a documented efficacy and safety profile in randomized clinical trials [4,5]. However, additional treatment strategies are desirable as hypothermia has limited effects and almost half of all infants treated with hypothermia still suffer permanent neurological impairment [6]. Therefore, there is an urgent need for novel additional or complementary therapeutic strategies to treat injury in the developing brain.

Emerging evidence suggests that inhibition of death-associated protein kinase 1 (DAPk1), which prevents excessive NMDA receptor (NMDAR) activation without interfering with physiological functions, provides neuroprotection in animal models of adult stroke [7,8]. The NMDAR and DAPks are particularly highly expressed in the immature brain, suggesting that DAPk1-mediated mechanisms may also play a role in the injured developing brain [9,10]. Shear stress of up to 6 h is reported to up-regulate and activate DAPk expression in endothelial cells [11]. This suggests that DAPk may also have a role in other types of brain injuries such as traumatic brain injury. This review aims to shed new light onto the structure of DAPks and DAPk1-mediated mechanisms and regulation of neuronal death, with special emphasis on injury in the developing brain.

## 2. NMDA Receptor/Excitotoxicity in the Developing Brain

Neonatal hypoxia-ischemia or direct excitotoxic insults induce a cascade of neurotoxic events in the brain [1] that lead to energy failure, glutamate release, activation of NMDA receptors, intracellular influx of calcium, and formation of nitric oxide. These changes result in mitochondrial dysfunction and leakage of O_2_ during reperfusion, which contribute to the formation of more reactive oxygen species that damage lipids, proteins and other cell components ultimately leading to cell death [12,13]. Despite promise in preclinical animal models [14,15], particularly in the immature brain, clinical trials for direct antagonism of the NMDAR as a therapeutic treatment have been disappointing, perhaps because of interference with physiological NMDAR function or a narrow therapeutic window.

However, mounting evidence suggests that these problems might be circumvented by selectively targeting molecular components of the death signalling cascade downstream of the NR2B subunit of NMDARs. The NR2B-containing NMDARs (NR2BRs) are preferentially localized at the extrasynaptic sites, which have been linked to cell death, but not with physiological functions [16,17]. Although DAPk-1 inhibitors are not expected to interfere with physiological functions their role in certain signalling pathways must be carefully considered. For example, DAPk has tumour suppressor properties and can selectively inhibit TCR [18] induced NFκB activation. The NR2BRs are of particular importance in brain development as they are the most abundant subunit in the immature brain [19], while with developmental maturation the synapses become increasingly populated by NR2A-containing NMDARs.

## 3. Death Associated Protein Kinases (DAPks)

The death-associated protein kinases include a family of calcium/calmodulin (Ca^2+/^CaM) dependent serine/threonine kinases whose relatives subsume DAPk1, DAPk-1 related protein 1 (DRP-1; DAPk-2), Zipper interacting protein kinase (ZIPk; DAPk-3), DAPk-1 related protein 1 (DRAk-1) and DRAk-2. DAPk1, DRP-1 and ZIP kinases are usually grouped into one superfamily since they share 83% and 80% identity at the amino acid level, respectively, with DAPk1’s catalytic domain, whereas DRAk-1 and DRAk-2 share 50% identity with DAPk1. Although DRP1 and ZIPk are not reported to be directly regulated in response to cerebral ischemia, DAPk1 mRNA levels are increased in response to neonatal hypoxia-ischemia *in vivo* [10].

DAPk1, ZIPk1 and DRP1 are proposed to form a unique hierarchy to activate cell death functions [20]. DRP1 is reported to be the upstream protein of all the DAPks and is involved in the activation of DAPk1 and ZIPk [21]. DAPk1 and ZIP kinase bind to each other via their catalytic domains phosphorylating ZIPk at six specific sites in the extra-catalytic *C*-terminal domain thereby leading to amplification of death-promoting signals [20]. Thus, these kinases form a “death-associated multi-protein complex” that translate, modulate and amplify various stress signals that lead to cell death. Since DAPk activation occurs from their linear interactions, it may seem that the inhibition of the upstream protein, DRP1, be sufficient to attenuate the cell death pathways induced by DAP kinases. However, due to the presence of multiple activating sources for these proteins, therapeutic strategies directed towards multiple targets have been suggested to yield better outcome [22].

## 4. Structure of DAPk Domains

**Figure 1:**
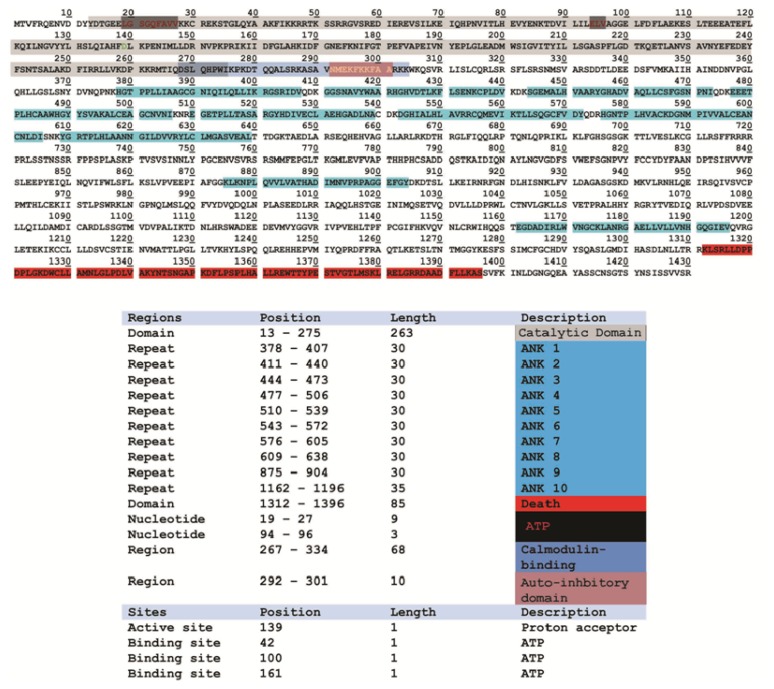
Amino acid sequence (single-letter amino acid code) for DAPk1 adapted from UniProt database. The structural and functional domains of DAPk1 and critical amino acid residues and domains are marked in the table.

DAPk1 is a 160-kD protein consisting of 1430 amino acids (Refseq: accession NM_004938.2). It consist of a protein kinase domain, a calcium-calmodulin binding region of 62 amino acids, 10 ankyrin repeats each about 30 amino acids in length, two putative P loops, cytoskeletal binding region and a death domain (Figure 1). More than 22 crystal structures of DAPk1, crystalized with their respective inhibitors at a resolution higher than 2.5 Angstroms, are reported in the Protein Data Bank (PDB). The crystal structure of DAPk1 is an excellent example of a small-molecule fragment bound to the kinase domain, which can be used as the starting point for bioavailable protein kinase inhibitor design, allowing *in vitro* and *in vivo* target validation studies to be performed. However, although initial target validation evidence with bioavailable kinase inhibitors supports DAPk1 as a drug discovery target for neurological disorders, no clinically promising small-molecule DAPk1 inhibitors have yet been discovered. Therefore, the development of small molecule inhibitors for DAPk1 is an attractive treatment option for perinatal brain injury since they have reduced adverse effects, can easily be administered and screened for specificity and capacity of binding with a target. To understand how the DAPk family of complex, multi-domain proteins operates in a cellular context, and how their dysfunction leads to disease, it is important to gain insight into how their individual domains relate to one another. For this purpose, we performed structural studies describing the precise spatial arrangement of DAPk1 domains as presented below.

### 4.1. The Catalytic Domain

**Figure 2:**
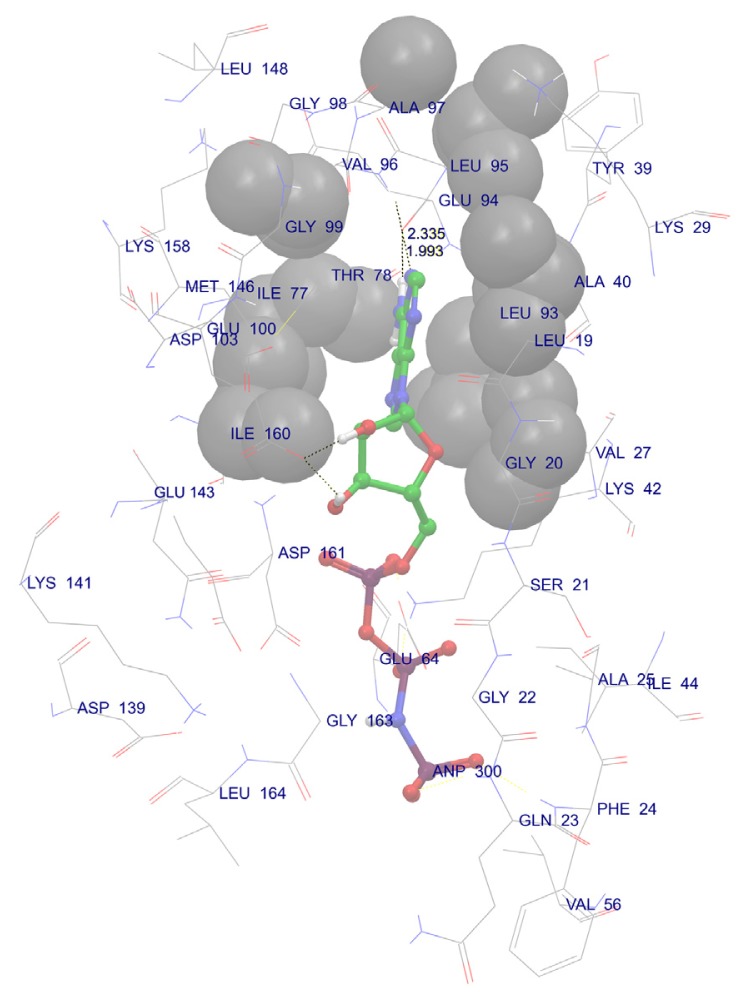
The Crystal structure of the catalytic domain of Dapk1 with docked ligand ANP (Phosphoaminophosphonic Acid-Adenylate Ester) showing important H-bond interactions (Black dotted lines). The grey spheres represent hydrophobic enclosures.

The catalytic domain of DAPk1 is composed of 11 subdomains, which have been implicated in many cellular functions [21]. The 3D coordinates of the X-ray crystallographic structure of human DAPk1 complex with respective inhibitor (PDB code: 1IG1) [23] were prepared by protein preparation wizard of Schrödinger (Schrödinger LLC, Portland, OR, USA) and all heteroatoms (except inhibitor) were removed from the protein file. The active site was analyzed by selecting neighbors within 5 Å around the respective ligand. All water molecules (3 Å far from inhibitor) were removed from the complex and the protein was minimized using OPLS-2005 force field. H-atoms were added to the protein to correct ionization and tautomeric states of amino acid residues. We removed the inhibitor from the active site of the DAPk and re-docked in to active site using glide [24] module of Schrödinger after preparing the ligand using LigPrep. We found that the active site of DAPk1 accommodates certain highly conserved amino acid residues such as Val96, Glu94, Glu100, Lys42, Phe24, Asp161 and Gly23, which are involved in H-bond interaction with their respective ligands and the hydrophobic interactions include Val27, Leu19, Ile160, Met146 and Ile77 residues. These interactions were in accordance with our active site analysis and also PDB data. Further we found additional hydrophobic interactions with Leu 93 and Ile 77. The analysis also revealed that the hydrogen bond interaction with Val 96 and Glu 94 are especially important since they reside in a hydrophobic enclosure (Figure 2).

### 4.2. The Calcium-Calmodulin Binding Region

DAPks are Ca^2+/^CaM-dependent kinases that are regulated by a double-locking mechanism. DAPk1 activity is regulated through several phosphorylation sites that are located within the CaM autoregulatory domain, two of which are Ser289 and Ser308. Full activation requires both the dephosphorylation of Ser308 and CaM binding. Binding of Ca^2+^ recruits CaM to the autoregulatory CaM-binding segment pulling this domain out from the catalytic cleft. Dephosphorylation of Ser308 increases the affinity for CaM thereby promoting the catalytic activity at low CaM levels. It has been shown that the deletion of the CaM-binding domain from DAPk or the substitution of Ser308 to Ala, generates a constitutively active kinase thereby exhibiting greater Ca^2+^ independent catalytic activity and killing potential [21,25]. The overall DAPk-CaM interface is almost 2000 Å^2^ in area [26].

**Figure 3:**
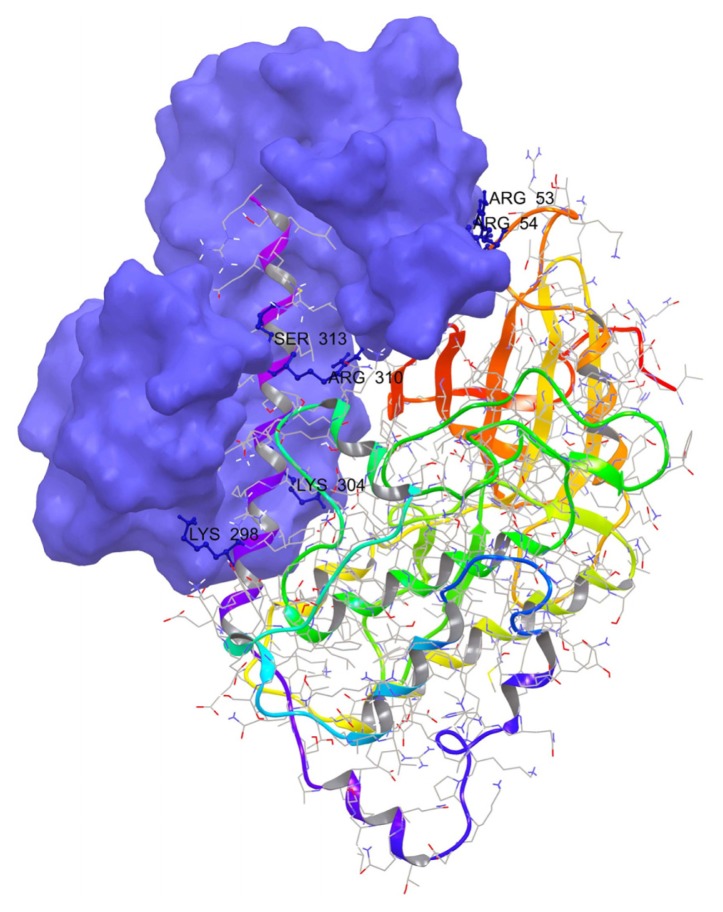
The Crystal structure of the Calcium-calmodulin binding domain showing the molecular basis of DAPK-CaM binding. CaM is shown in violet and DAPk1 is shown as ribbons. Residues of DAPk1 involved in specific interactions with calmodulin are labeled in blue.

In order to further analyze the CaM binding region we retrieved, from the crystal structure of the binary DAPK-CaM complex, the DAPK catalytic domain and adjacent autoregulatory domain complexed with CaM from PDB (PDB code: 1IG1) [26] and critically analyzed the structures. Protein structures were prepared in the same way as stated in section 4.1. The observed CaM interactions with the DAPk catalytic domain were restricted to the first helix of the N-terminal CaM lobe, two residues (Arg 53, Arg 54) of the DAPk family–specific basic loop, and two additional residues (Arg 23, Lys 222) from other Catalytic Domain surface loops. We found that the main interacting residues in the CaM-binding domain are Lys304, Ser308, Lys298 and Arg310 (Figure 3).

### 4.3. The Death Domain and Ankyrin Repeats

The death domain of DAPk1 is largely associated with protein-protein interactions, kinase activity and apoptotic functions. Deletion of the death domain is reported to abrogate the apoptotic functions of the kinase including TNF-α- and Fas-induced cell death [25]. Binding studies using death domain mini-proteins *in vitro* and deletion analysis *in vivo* determined that the death domain of DAPk1 is the major site for the interaction with tumor suppressor protein tuberin (TSC2). Thus, there is a positive association between growth factor stimulation of DAPk and mTORC1 signaling, which may ultimately affect autophagy, cell survival, or apoptosis [27]. Further, death domain-containing proteins have links to innate immunity, communicating with Toll-like receptors through bipartite adapter proteins such as MyD88 [28].

**Figure 4:**
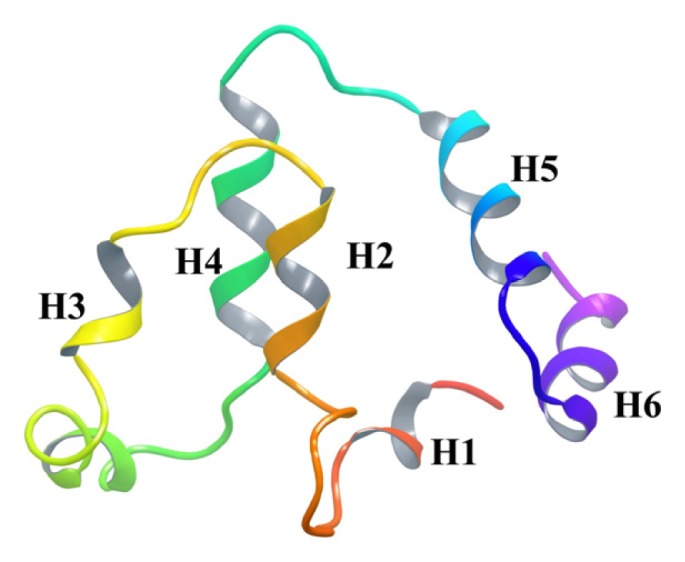
Ribbon representation of a homology model of the DAPk1 death domain. Alpha helices are labelled from H1 to H6.

The death domain is the least studied domain of DAPk1 and no crystal structure has been reported so far. In order to identify structural features, we used bioinformatics and cheminformatics tools to build homology models of the death domain of the DAPk1 protein. I-TASSER web server was used for structure prediction, which combines the methods of threading, *ab initio* modeling and structural refinement [29,30]. The exact interacting amino acid residues in the death domain responsible for interactions with interacting partners have not yet been defined. The homology model described here can serve as a template for future protein-protein interaction studies and also provides insights into the binding mode of the proteins (Figure 4).

**Figure 5:**
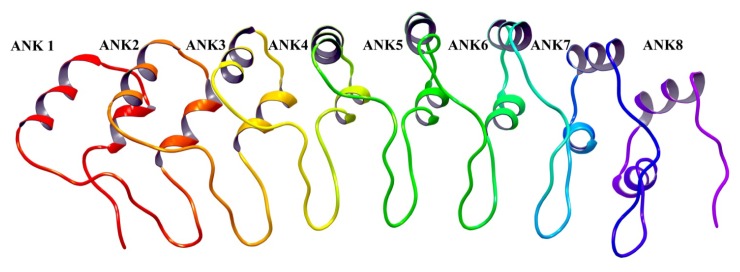
The homology model ankyrin repeat domain of DAPk1 is depicted as a ribbon structure.

The ankyrin repeats facilitate protein-protein communications and are implicated mostly in DAPk1 degradation. s-DAPk-1, an alternatively spliced product lacking the DAPk1 kinase domain regulates the steady-state levels of the full-length DAPk-1 and is implicated in proteasome-independent degradation pathway for DAPk1 [31] The ankyrin repeats also facilitate the DAPk1 degradation via the ubiquitin-proteasome pathway. The E3 ubiquitin ligase DIP-1 (MIB-1) interacts with the ankyrin repeats and is able to actively ubiquitinate and degrade DAPk1 [32]. As DAPk interacts with HSP90 through its kinase domain, the E3 ubiquitin ligase carboxyl terminus of HSC70-interacting protein (CHIP), which facilitates the ubiquitination of HSP90-interacting proteins, will also induce DAPk1 degradation [33,34]. The crystal structure of the ankyrin repeats of DAPk1 has not been described and we have used similar approaches as above to develop a homology model of ankyrin repeats. As the ankyrin repeat of DAPk1is a protein–protein interaction domain the homology model developed can be used for protein-protein interaction studies, and identification of protein hotspots, which represent the first step in rational drug design projects (Figure 5).

## 5. Interacting Partners of DAPk1

As described above, DAPk1 is a relatively large protein with multiple domains and docking motifs that drive its regulation and function. Understanding protein interactions at the domain level gives a global view of the protein interaction network, and possibly of protein functions. However, to date only certain domains of the DAPk1 protein have been purified by crystallographic studies and the native substrates of DAPk and the molecular pathways underlying DAPk-mediated signal transduction still remain unclear to a large extent. Biochemical studies demonstrate that DAPk1 binds syntaxin-1 via their *C*-terminal domains and phosphorylates it at Ser188. This phosphorylation event occurs both *in vitro* and *in vivo* in a Ca^2+^-dependent manner [35]. PDCD6, a well-recognized apoptotic mediator, has been identified as a binding partner of DAPk1. Co-transfection of PDCD6 and DAPk1 cDNA into a tumour cell line accelerated apoptosis via the caspase-3 dependent pathway [36].

DAPk1 has been found to interact with ERK1 and ERK2 through a specific docking sequence within its death domain. Phosphorylation of DAPk1 at Ser735 by ERK2 increases the catalytic activity of DAPk1 both *in vitro* and *in vivo*. DAPk1 promotes the cytoplasmic retention of ERK1/2, thereby inhibiting ERK signaling in the nucleus ultimately promoting the apoptotic activity of DAPk1 [37]. Further, DAPk1 plays a leading role in oxidative stress-induced JNK signaling via protein kinase D (PKD), which has been identified as a novel substrate of DAPk1 [38]. It was also found that small linear peptide interaction sites or docking motifs play an important role in DAPk1 function. Immunobinding assays demonstrated that DAPk1 can bind to the full-length human MAP1B, thereby stimulating membrane blebbing and autophagy [39]. DANGER, a novel protein identified on the basis of its binding to inositol 1, 4, 5-trisphosphate receptors (IP3R) protein, contains a partial MAB-21 domain. The direct binding of DANGER to DAPk1 inhibits DAPk1 catalytic activity, and DANGER knock-out mice exhibit augmented neuronal and non-neuronal cell death both *in vivo* and *in vitro* [39,40]. Thus, DANGER appears to regulate cell death physiologically through its inhibition of DAPk1 signaling. DAPk1 is also responsible for the phosphorylation of Pin1 on Ser71 in its catalytic active site. Such phosphorylation fully inactivates Pin1 catalytic activity and inhibits its nuclear location and cellular function [41].

## 6. DAPk-Regulation in NMDA-Induced Cerebral Injury

Much of our knowledge regarding the mechanisms of hypoxic-ischemic brain damage, as well as potential therapeutic interventions, evolves from literature on experimental adult stroke models. However, there is a distinct difference to the damaging effects of hypoxia-ischemia between the adult and the neonatal brain. In some respects, the immature brain is more resistant to hypoxic-ischemic damage than its adult counterpart, but at the same time exhibiting periods of heightened sensitivity to injury, dependent on the specific developmental stage of the brain [42]. Importantly, there is now ample evidence to show that cell death pathways differ between the immature and adult brain [43,44]. In the immature brain, NMDA channels open more easily and remain open longer than in the adult brain, and the voltage-dependent magnesium block that is normally present in adult channels at resting membrane potentials is more easily relieved in the perinatal period [45]. One of the most significant developmental differences in the response to hypoxia-ischemia is that apoptotic mechanisms are much more prominent in the immature brain compared with older brains [43]. Thus it is of utmost importance that different and specific therapeutic paradigms are considered for the adult and immature brain.

**Figure 6:**
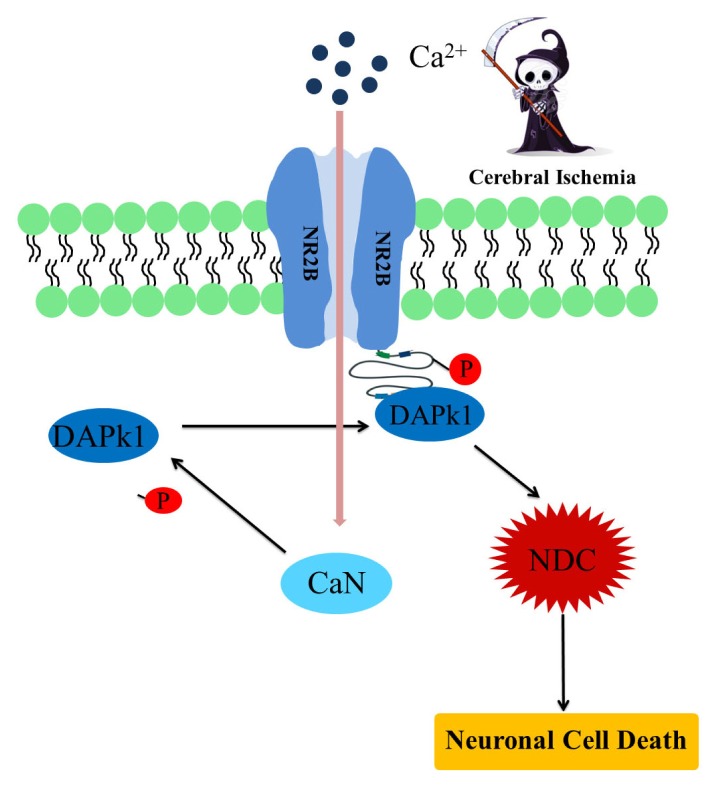
Proposed DAPk1-mediated cell death pathway. Cerebral ischemia results in Ca^2+^ influx through the NMDA receptor channel, which activates calcineurin (CaN) and dephosphorylates DAPk1 at pS308. This leads to activation of DAPk1, which in turn phosphorylates the NR2B subunit of the NMDA receptor. DAPk1 recruitment then promotes the activation of death-signaling proteins in a neuronal death signaling complex (NDC). The NDC include all neuronal death-signaling proteins that closely associate with the NMDAR channel pore like the NR2B–PSD95–nNOS signaling complex.

The activity of the NMDAR-ion channel complex is tightly regulated by a number of pharmacologically distinct binding sites [46]. However, under pathological conditions, such as cerebral ischemia, the regulation of the NMDAR activity fails resulting in an excessive cellular influx of calcium, which leads to cell death. Dysregulation of the NMDAR following cerebral ischemia in adult mice was recently linked to recruitment of DAPk1 into the NR2BR protein complex. It was shown that DAPk1 physically and functionally interacts specifically with the NMDA receptor NR2B subunit at extra-synaptic sites, which has been identified as a central interaction site that mediates stroke damage [7]. The fact that NR2BRs are increasingly populated in the immature brain [19] leads us to the conclusion that DAPk1 is an important novel pharmacological target for intervention in neonates. DAPk1 is inactive in normal brain tissues, where it is found in its phosphorylated state. It becomes rapidly and persistently dephosphorylated and activated in response to ischemia *in vivo* and directly binds with the NMDA receptor NR2B *C*-terminal tail, at amino acids 1292–1304. Subsequently DAPk1 phosphorylates the NR2B subunit at Ser1303 and enhances the NR1/NR2B receptor channel conductance resulting in excessive Ca^2+^ influx through the receptor channels thereby triggering a cascade of intracellular events that arbitrate cell death. It has been suggested that DAPk1 potentiates NR2BR activity, thereby promoting neuronal death by activation of “calcium-activated death-signaling proteins like the NR2B–PSD95–nNOS signaling complex” [17]. Under pathological conditions such as ischemic insults Ca^2+^ influx through NMDAR channels activates calcineurin (CaN), which in turn dephosphorylates DAPk1 at pS308, leading to its activation thereby acting as an upstream activator of DAPk1 (Figure 6). It has also been reported that dephosphorylation of p-S308 in response to ischemic insults can be eliminated by genetic mutation of CaN [7].

In support of a role for DAPk1 in brain injury, a selective DAPk inhibitor was shown to be neuroprotective in both *in vitro* and *in vivo* ischemic models [7,8]. In addition, treatment of rats with a small-molecule DAPk1 inhibitor even 6 hours after cerebral ischemia attenuated the loss of brain tissue, measured one week later [47,48]. Thus inhibition of DAPk1 prevents the over-stimulation of NMDARs following stroke damage, without interfering with its physiological functions.

## 7. Novel Approaches to Blocking the Effects of DAPk1

With the evidence of DAPk1-mediated injury in cerebral ischemia and the ability of bioavailable DAPk inhibitors to rescue neuronal death, DAPk1 has emerged as an important drug-discovery target for brain disorders. Thus, by identifying the binding domains and exact interacting residues of DAPk1 we can potentially efficiently disrupt the interaction of DAPk1 with its partners, thus ameliorating brain injury. Some examples of novel, potentially neuroprotective, strategies are outlined below.

A strong interest exists for ATP-competitive inhibitors, which bind to the active site within the catalytic domain that is required for the functions of DAPk complexes, resulting in down regulation of death signaling globally [49]. However, as with many other protein kinase inhibitors, some DAPk inhibitors are likely to non-specifically inhibit multiple kinases at therapeutic concentrations. This raises concerns over whether the therapeutic activity of some compounds was due to specific proposed target inhibition.

Compounds that act outside the ATP-binding pocket are also of considerable interest. As discussed above, DAPk1 and DAPk2 are CaM-dependent kinases and disrupting the CaM-DAPk1 interaction by means of small molecules may be an effective mechanism to inhibit the protein. The death domain is a prime mediator of the interactions necessary for transducing a death signal. Therefore, silencers of the death domain, such as the SODD/BAG-4-related proteins, could be potential therapeutic agents [50]. At this point, no structural basis exists of how conformational changes affect death domain interactions and how they are propagated to the remainder of the protein. One important goal that remains to be achieved is a level of understanding that would enable the rational design of small molecules capable of modifying the interactions between specific members of this group [51].

Cathepsin B is another protein with a novel binding domain localized in the *C*-terminal domain of DAPk1 between the ankyrin repeats and the death domain. DAPk1 forms a multi-protein survival complex with cathepsin B countering the rate of TNFR-1-dependent apoptosis and highlights the importance of developing DAPk1 inhibitors as agents to sensitize cells to stress-induced apoptosis [52] siRNA-based therapeutics with the ability to silence expression of DAPk1 is also currently under investigation. TNF-α or INF-γ-dependent NF-κB activity was enhanced by the inhibition of DAPk1 with its specific siRNA leading to cell survival [53]. Similarly down-regulation of DAPk1 by transfecting siRNA in Jurkat cells resulted in reduced susceptibility to Fas-induced apoptosis suggesting the involvement of DAPk1 in the extrinsic apoptosis pathway in lymphocytes [54]. The s-DAPk1 is derived from a small internal mRNA from the DAPk1 locus which can mimic full-length DAPK1’s ability to promote membrane blebbing. Cleavage of the *C*-terminal tail of s-DAPk1 can regulate the ability of the protein to mimic the biological functions of DAPk1 in promoting membrane blebbing and controlling the half-life of DAPk1 protein itself. Therefore s-DAPk could be exploited to inhibit the DAPk1 functions [52,55].

Further, kinase targets are usually components of complex, interconnected signal transduction cascades comprising many protein kinases, with pathway redundancy and crosstalk between pathways [56]. Cross talk between major subcellular organelles suggests that therapeutic strategies should be optimally directed at multiple targets or pathways for better therapeutic outcome [57]. The interaction between DAPk1 and the NMDA-receptor subunit NR2B is at the same site that also interacts with CaMkII. This raises the possibility that some of the neuroprotective interventions designed to target DAPk1 or CaMkII may act in part through effects on the other kinase and therefore targeting both kinases, with a single compound, may be desirable for maximal therapeutic effect [56].

## 8. Conclusions

Neonatal hypoxia-ischemia triggers a cascade of biochemical and molecular events, including excitotoxicity through the NMDA receptor. Brain damage is progressive and evidence suggests a distinct difference between the immature and mature brain in the pathology and consequences of brain injury [58]. Thus, evidence from experiments in adult animals cannot easily be translated to the neonatal situation and it is important to investigate neuroprotective strategies in appropriate animal models that reflect the difference in brain development. DAPk mRNA is markedly increased in brain tissue homogenates following neonatal hypoxia-ischemia. However, there is presently little information on the role of DAPk1 activation in the developing brain. Understanding the mechanisms behind the complex molecular cascades of injury in neonates will provide insight required to develop novel strategies to prevent or avert the long-term consequences of neonatal brain injury, such as cerebral palsy and epilepsy.
